# Utilisation of mobile X‐ray services by residents of long‐term care facilities

**DOI:** 10.1111/imj.70034

**Published:** 2025-03-24

**Authors:** Maria C. Inacio, Robert N. Jorissen, Virginie Gaget, David R. Tivey, Joanne Dollard, Renuka Visvanathan, Guy J. Maddern

**Affiliations:** ^1^ Registry of Senior Australians South Australian Health and Medical Research Institute Adelaide South Australia Australia; ^2^ Allied Health and Human Performance Academic Unit University of South Australia Adelaide South Australia Australia; ^3^ Discipline of Surgery University of Adelaide, The Queen Elizabeth Hospital Woodville South Australia Australia; ^4^ Royal Australasian College of Surgeons Adelaide South Australia Australia; ^5^ Adelaide Geriatrics Training and Research with Aged Care Centre (GTRAC), Faculty of Health and Medical Sciences University of Adelaide Adelaide South Australia Australia; ^6^ Aged & Extended Care Services The Queen Elizabeth Hospital and Basil Hetzel Institute, Central Adelaide Local Health Network Adelaide South Australia Australia

**Keywords:** nursing home, residential facility, X‐ray, mobile health unit, diagnostic imaging

## Abstract

**Background:**

Access to mobile X‐ray imaging (MXR) by long‐term care facility (LTCF) residents could potentially reduce emergency department transfers. To encourage MXR use, the Australian Government Medicare Benefits Schedule introduced an MXR service subsidy in November 2019.

**Aims:**

To examine the (i) MXR utilisation rate in LTCFs between 1 November 2019 and 30 June 2020; and (ii) individual and LTCF characteristics associated with accessing MXR compared to community‐based X‐rays.

**Methods:**

A cross‐sectional study of non‐indigenous LTCF residents, ≥65 years old, between 1 November 2019 and 30 June 2020 in five geographical regions was conducted. Access to mobile and community‐based X‐rays was the outcome of interest. Descriptive statistics and monthly sex‐ and age‐standardised utilisation rates were employed. Characteristics associated with the X‐ray type accessed were estimated using generalised estimating equation logistic regression models.

**Results:**

149 389 LTCF episodes, by 127 002 individuals, in 1449 facilities were studied. The median age of the studied individuals was 85 years (interquartile range 79–90) and 63.4% (*n* = 94 692) were women. There were 5458 (3.7%) episodes that accessed an MXR service. MXR usage increased 75%, from 6.6/1000 (95% confidence interval (CI) 6.1–7.2) to 11.6/1000 (95% CI 10.9–12.3) person‐months over the study period. Compared to community‐based X‐ray recipients, MXR recipients were older, more likely to have dementia, but less likely to have a higher number of health conditions, be in transition or respite care, be in a not‐for‐profit LTCF and be outside a major city.

**Conclusions:**

A small but increasing use of government‐subsidised MXR services was observed. Individuals accessing MXRs are those that likely benefit most from them. LTCF differences in service utilisation indicate gaps in service access.

## Introduction

The world's population is ageing, and demand for high‐quality integrated social and healthcare is increasing.^1^ In Australia, approximately 246 000 people are permanent residents of long‐term care facilities (LTCF) yearly,^2^ which is projected to increase in volume until 2038, despite a decrease in utilisation rate.^3^ Compared to those living in the community supported by other types of care, individuals entering LTCFs generally are older and have poorer health, higher frailty and a higher prevalence of dementia.^4^ These individuals also have a high risk of emergency department presentations and unplanned hospitalisations, often for falls, respiratory conditions and fractures.^5^, ^6^, ^7^, ^8^ Healthcare services and technology that can facilitate care access and integration while avoiding hospitalisations in individuals in LTCFs offer potential benefits to older residents. This includes access to mobile X‐ray imaging, which plays a critical role in the diagnosis and management of many medical conditions affecting LTCF residents for which they are often hospitalised.

Mobile X‐ray imaging, and mobile radiology in general, allows imaging to be performed outside a hospital or clinical setting. For LTCF residents, this can avoid the inconvenience of transfers to hospital and risks of negative hospitalisation‐related events (e.g. delirium, hospital deconditioning) and hospital congestion.^9^, ^10^ Additionally, the national utilisation of plain X‐ray services by residents of LTCFs increased up to 2016, which was likely related to the rising frailty and higher number of health conditions these individuals are experiencing at entry to LTCFs.^11^ To encourage mobile X‐ray service use in Australia, the Australian Government Medicare Benefits Schedule (MBS) introduced a subsidy to support the reimbursement of mobile X‐ray services nationally. MBS item 57 541 was introduced on 1 November 2019 to partly reimburse the transport of the X‐ray machine to the patient.^12^ Claims for this MBS item are limited to the first eligible X‐ray procedure performed at a LTCF, provided the service had been requested by a medical practitioner who had personally seen the intended recipient. The subsidised service is also restricted to plain X‐ray imaging use for the specific diagnosis of fall‐related injuries, suspected pneumonia, heart failure, acute abdomen or bowel obstruction (Table S1).

To date, an evaluation of the uptake of the MBS subsidy for mobile X‐rays in its initial 17 months of implementation has shown that by March 2021 about 9.5 services/1000 residents of LTCFs were used nationally.^11^ However, the examination of the profile of residents and LTCFs accessing these services is yet to be conducted. This evaluation can inform government, X‐ray service providers and LTCF providers of the accessibility and reach of these services nationally and contribute to the identification of gaps in reach. Specifically, our study examined the (i) utilisation rate of mobile X‐ray by resident and LTCF characteristics between November 2019 and June 2020 and (ii) resident and LTCF characteristics associated with accessing mobile X‐ray compared to X‐rays accessed in the community.

## Methods

### Study design, setting and data source

A cross‐sectional study using the Registry of Senior Australians (ROSA) National Historical Cohort was conducted.^13^ ROSA contains de‐identified, integrated information from the aged care, healthcare and social welfare sectors from national and state‐based data collections for individuals that have entered the aged care sector in Australia. To date, the 2002–2020 National Historical Cohort includes information on almost 3.5 million older people who have accessed any aged care services.^13^ In this study, the following data collections from ROSA were used: aged care eligibility assessments (collected using the National Assessment Screening Form), residential aged care service records (episodes of care), entry into residential aged care needs assessments (collected using the Aged Care Funding Instrument), Australian Government MBS and Pharmaceutical Benefits Scheme datasets, and Australian Institute of Health and Welfare's National Death Index.

### Study cohort

The study cohort included non‐Indigenous residents ≥65 years old who lived in a LTCF for at least 1 day between 1 November 2019 (introduction of MBS item 57 541) and 30 June 2020 (latest data available) in five geographical regions with mobile X‐ray providers nationally. These regions include Greater Sydney (New South Wales, NSW), Illawarra and Shoalhaven (NSW), greater Melbourne (Victoria), Greater Brisbane and Gold Coast regions (Queensland) and Greater Adelaide and Fleurieu–Kangaroo Island regions (South Australia). Individual episodes at a LTCF were the unit of analysis, as some individuals had more than one episode (149 389 episodes by 127 002 individuals).

### Outcome of interest

The primary outcome of interest was the reception of a MBS‐subsidised (item 57 541) mobile X‐ray service. Only X‐rays for suspected fall‐related injuries, pneumonia, heart failure, acute abdomen or bowel obstruction, which are eligible for the mobile X‐rays subsidy, were examined (Table S1 shows MBS items for specific X‐rays). This outcome was ascertained from both recorded 57 541 items or inferred from 57 541‐eligible X‐ray service items from one of the (de‐)identified providers of mobile X‐ray services concurrent with a date and LTCF code of a 57 541 item. An additional outcome of interest was the provision of X‐ray services for the same indications eligible for mobile X‐ray (Table S1), categorised by whether the X‐rays were mobile services or delivered in the community.

### Exposures of interest

Individuals’ covariates examined included age, sex, number of health conditions (6 months look‐back from cohort entry),^14^ dementia status^15^ and LTCF service type (permanent, respite or transition care). Facility characteristics examined included ownership (for‐profit, not‐for‐profit and government), state, geographical area and average resident cohort size (0–24, 25–49, 50–74, 75–99 and 100+ beds) during the study period.

### Statistical analysis

Descriptive statistics were used to characterise the study cohort. Sex‐ and age‐standardised (directly) utilisation rates of mobile radiology services per 1000 person‐(30‐day) months and 95% confidence intervals (CI) were estimated by month. Stratification of utilisation rates by covariate was examined. Differences in resident and LTCF characteristics using mobile X‐ray services compared to community X‐ray services were evaluated using a generalised estimating equation logistic regression model, with a random effect for LTCF and an exchangeable correlation matrix. Odds ratios and 95% CIs are presented. Missing data (*n* = 1760, 6.1%) were managed via case‐wise deletion, and a sensitivity analysis was performed in which the regression was repeated without the dementia variable, which had the most missing values (missing data = 59, 0.2%). All tests were two‐sided, and α < 0.05 was considered statistically significant. Data were analysed using R version 4.0.3 and SAS version 9.4.

### Ethics

This study received ethics approvals from the University of South Australia (ID:200489) and the Australian Institute of Health and Welfare (reference: EO2022/4/1376).

## Results

### Study cohort

The cohort included 149 389 LTCF episodes by 127 002 individuals in 1449 LTCFs. The median length of follow‐up for residents’ episodes included was 243 days (IQR 47–243 days). The median age of the cohort was 85 years (IQR 79–90), 63.4% (*n* = 94 692) were women, and 53.4% (*n* = 79 759) had dementia (Table 1). Most of the episodes were for permanent care residents (76.1%, *n* = 113 623, median follow‐up 243 days, IQR 166–243), followed by respite care (19.5%, *n* = 29 087, median follow‐up 20 days, IQR 13–34) and transition care residents (4.5%, *n* = 6679, median follow‐up 40 days, IQR 20–70). In the cohort, 47.4% (*n* = 70 857) of the episodes occurred in not‐for‐profit, 47.0% (*n* = 70 239) in private and 5.6% (*n* = 8293) in government LTCFs (Table 1).

**Table 1:** Individual and long‐term care facility characteristics by use of mobile or community‐based X‐ray service use between November 2019 and June 2020†

	Total cohort		No X‐rays		Community X‐ray only		Mobile X‐ray‡	
	*n*	%	*n*	%	*n*	%	*n*	%
Total	149 389	100.0	129 568	86.7	14 363	9.6	5458	3.7
Resident characteristics								
Age								
0–74	18 568	12.4	16 457	12.7	1637	11.4	474	8.7
75–84	49 399	33.1	43 063	33.2	4733	33.0	1603	29.4
85–94	70 616	47.3	60 647	46.8	7066	49.2	2903	53.2
95+	10 806	7.2	9401	7.3	927	6.5	478	8.8
Sex								
Women	94 692	63.4	81 654	63.0	9386	65.3	3652	66.9
Men	54 697	36.6	47 914	37.0	4977	34.7	1806	33.1
Rx‐risk comorbidities								
0–3	43 484	29.1	39 468	30.5	2735	19.0	1281	23.5
4–6	63 479	42.5	55 152	42.6	6025	41.9	2302	42.2
7+	42 426	28.4	34 948	27.0	5603	39.0	1875	34.4
Dementia status								
Yes	79 759	53.4	70 396	54.3	6326	44.0	3037	55.6
Missing	14 135	9.5	12 703	9.8	1318	9.2	114	2.1
Facility characteristics								
Type of episode								
Permanent	113 623	76.1	96 388	74.4	12 103	84.3	5132	94.0
Respite	29 087	19.5	27 510	21.2	1268	8.8	309	5.7
Transition	6679	4.5	5670	4.4	992	6.9	17	0.3
Ownership								
Not‐for‐profit	70 857	47.4	61 595	47.5	6589	45.9	2673	49.0
Private	70 239	47.0	60 873	47.0	6633	46.2	2733	50.1
Government	8293	5.6	7100	5.5	1141	7.9	52	1.0
Size								
0–24	808	0.5	683	0.5	105	0.7	20	0.4
25–49	14 794	9.9	12 809	9.9	1474	10.3	511	9.4
50–74	30 573	20.5	26 732	20.6	2772	19.3	1069	19.6
75–99	34 417	23.0	30 093	23.2	3401	23.7	923	16.9
100+	68 797	46.1	59 251	45.7	6611	46.0	2935	53.8
Geographical area								
New South Wales – Greater Sydney	49 255	33.0	40 654	31.4	5035	35.1	3566	65.3
New South Wales – Other	5070	3.4	4426	3.4	509	3.5	135	2.5
Victoria	46 999	31.5	41 436	32.0	4396	30.6	1167	21.4
Queensland	27 775	18.6	24 802	19.1	2664	18.5	309	5.7
South Australia	20 290	13.6	18 250	14.1	1759	12.2	281	5.1
Remoteness								
Major city	143 268	95.9	124 094	95.8	13 755	95.8	5419	99.3
Inner regional	5935	4.0	5322	4.1	584	4.1	29	0.5
Remote	14	0.0	14	0.0	0	0.0	0	0.0
Missing	172	0.1	138	0.1	24	0.2	10	0.2

† Episodes of care is the unit of analysis. There were 149 389 episodes in long‐term care facilities by 127 002 individuals.

‡ Includes individuals who also received non‐mobile X‐rays, that is, X‐ray services in the community.

Mobile X‐ray services were used in 5458 (3.7%) LTCF episodes by 5429 individuals (4.3%), with 4788 (3.8%) of these using the service once, 540 (0.4%) using it twice and 101 individuals (0.1%) using it at least three times. During the same period, 14 363 (9.6%) episodes of LTCF received only non‐mobile X‐ray services in the community. There were 6195 mobile X‐rays provided for those in our cohort, which accounted for 21.5% of 28 861 eligible provisions of X‐ray services. Of these, 2761 (44.6%) were for fall‐related injuries, 2452 (39.6%) for pneumonia/heart failure and 475 (7.7%) for both (Table 2). The remaining were for the diagnosis of acute abdomen/bowel obstruction alone (6.3%, *n* = 391) or in combination with other types (1.9%, *n* = 116). The main body regions imaged were chest (44.6%, *n* = 2764), legs (16.1%, *n* = 999) and gastrointestinal tract/abdomen (11.6%, *n* = 717) (Table 2).

**Table 2:** Types of mobile X‐ray services received†

X‐rays groups	*n*	%
Mobile X‐rays eligible classes	6195	100.0
Fall	2761	44.6
Pneumonia/heart failure (HF)	2452	39.6
Fall; pneumonia/heart failure	475	7.7
Acute abdomen/bowel obstruction	391	6.3
Pneumonia/HF; acute abdomen/bowel obstruction	96	1.6
Fall; pneumonia/HF; acute abdomen/bowel obstruction	12	0.2
Fall; acute abdomen/bowel obstruction	8	0.1
Body region imaged		
Chest	2764	44.6
Legs	999	16.1
GI tract/abdomen	717	11.6
Upper body	617	10.0
Hip/pelvis	389	6.3
Upper body; GI tract/abdomen	165	2.7
GI tract/abdomen; head	148	2.4
Chest; hip/pelvis	100	1.6
Chest; legs	55	0.9
Legs; GI tract/abdomen	45	0.7
Chest; GI tract/abdomen	37	0.6
Chest; head	31	0.5
Legs; upper body	25	0.4
Chest; upper body	17	0.3
Chest; upper body; GI tract/abdomen	13	0.2
Legs; head	10	0.2
Upper body; GI tract/ abdomen; head	8	0.1
Chest; GI tract/abdomen; head	7	0.1
Other‡	48	0.8

† Some individuals had multiple X‐rays.

‡ ‘Other’ includes combinations with less than five individuals.

GI, gastrointestinal.

### Utilisation rate of mobile X‐ray services

**Figure 1:**
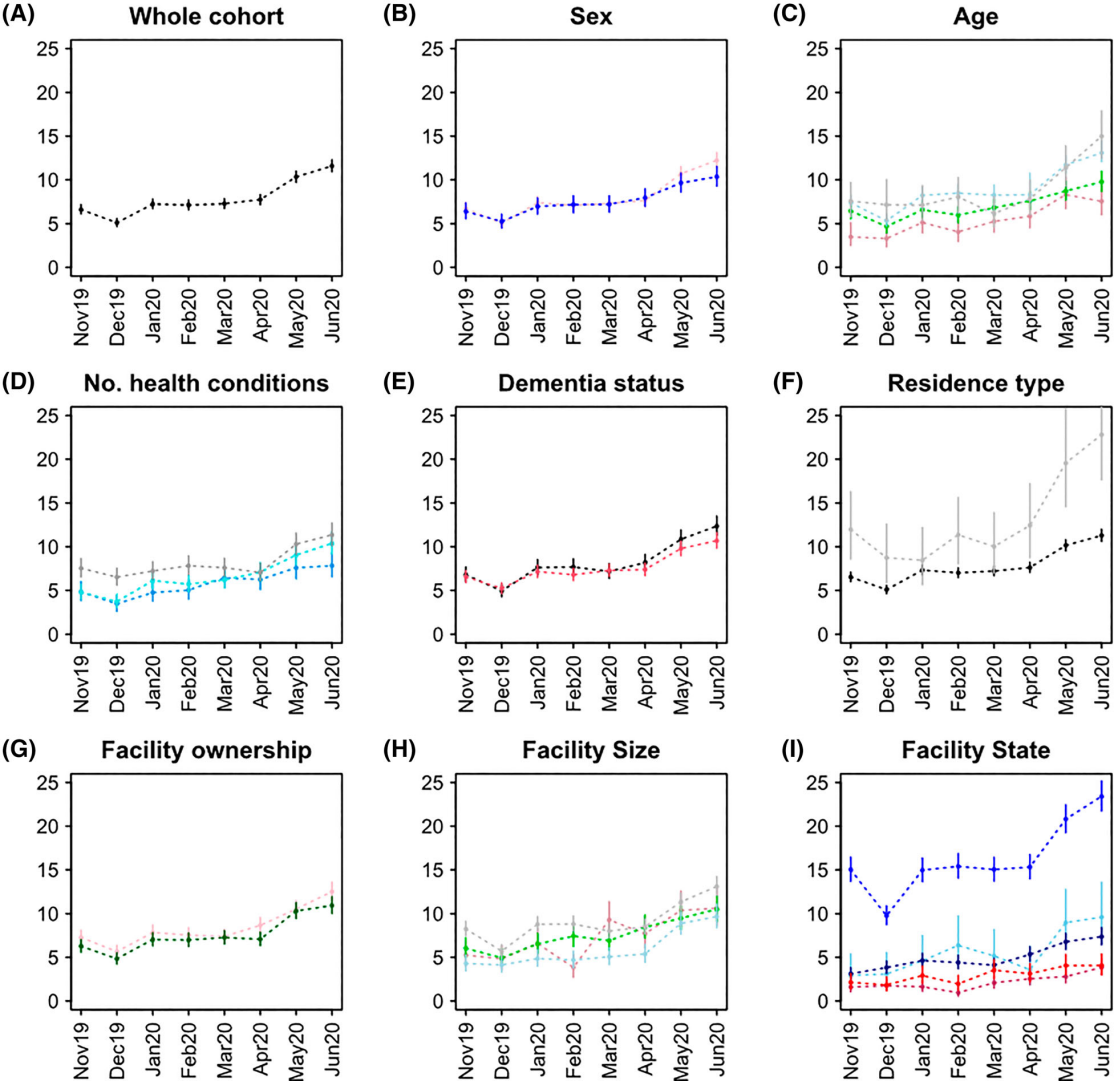
Sex‐ and age‐standardised mobile X‐ray service utilisation by residents of long‐term care facilities per 1000 person‐months, November 2019–June 2020. (A) Whole cohort; (B) sex: females (pink) and males (blue); (C) age, year groups: <75 years (pink), 75–84 (green), 85–94 (light blue) and 95+ (grey); (D) Rx‐Risk‐V health conditions: 0–3 (blue), 4–6 (light blue) and 7+ (grey); (E) dementia status: no dementia (black) and dementia (red); (F) LTCF residence type: permanent (black) and respite (grey) (transitional care not shown due to small numbers); (G) facility ownership: not‐for‐profit (black) and for‐profit (pink) (government facilities not shown due to small numbers); (H) facility size, places: 0–49 (pink), 50–74 (green), 75–99 (light blue), and 100+ (grey); (I) facility state/region: New South Wales (NSW) – Sydney (blue), NSW – other (light blue), Victoria (dark blue), Queensland (maroon) and South Australia (red).

Overall, there was a 75% (95% CI 59–93) increase in sex‐ and age‐adjusted utilisation of mobile X‐ray services over the study period: from 6.6/1000 (95% CI 6.1–7.2) in November 2019 to 11.6/1000 (95% CI 10.9–12.3) person‐months in June 2020 (Fig. 1). The greatest increase was observed between April (7.7/1000 person‐months, 95% CI 7.2–8.3) and June 2020. Similar increases were observed across sexes, dementia status and LTCF ownership type (excluding government LTCFs that had too few to examine). From April to June 2020, noticeable increases were observed in mobile X‐ray use by residents in respite care (from 12.0/1000 person‐months, 95% CI 8.6–16.3 to 22.8/1000, 95% CI 17.7–29.0) and residing in Greater Sydney (from 15.3/1000 person‐months, 95% CI 14.0–16.7 to 23.4, 95% CI 21.7–25.2).

### Factors associated with mobile versus community‐based X‐rays

**Figure 2:**
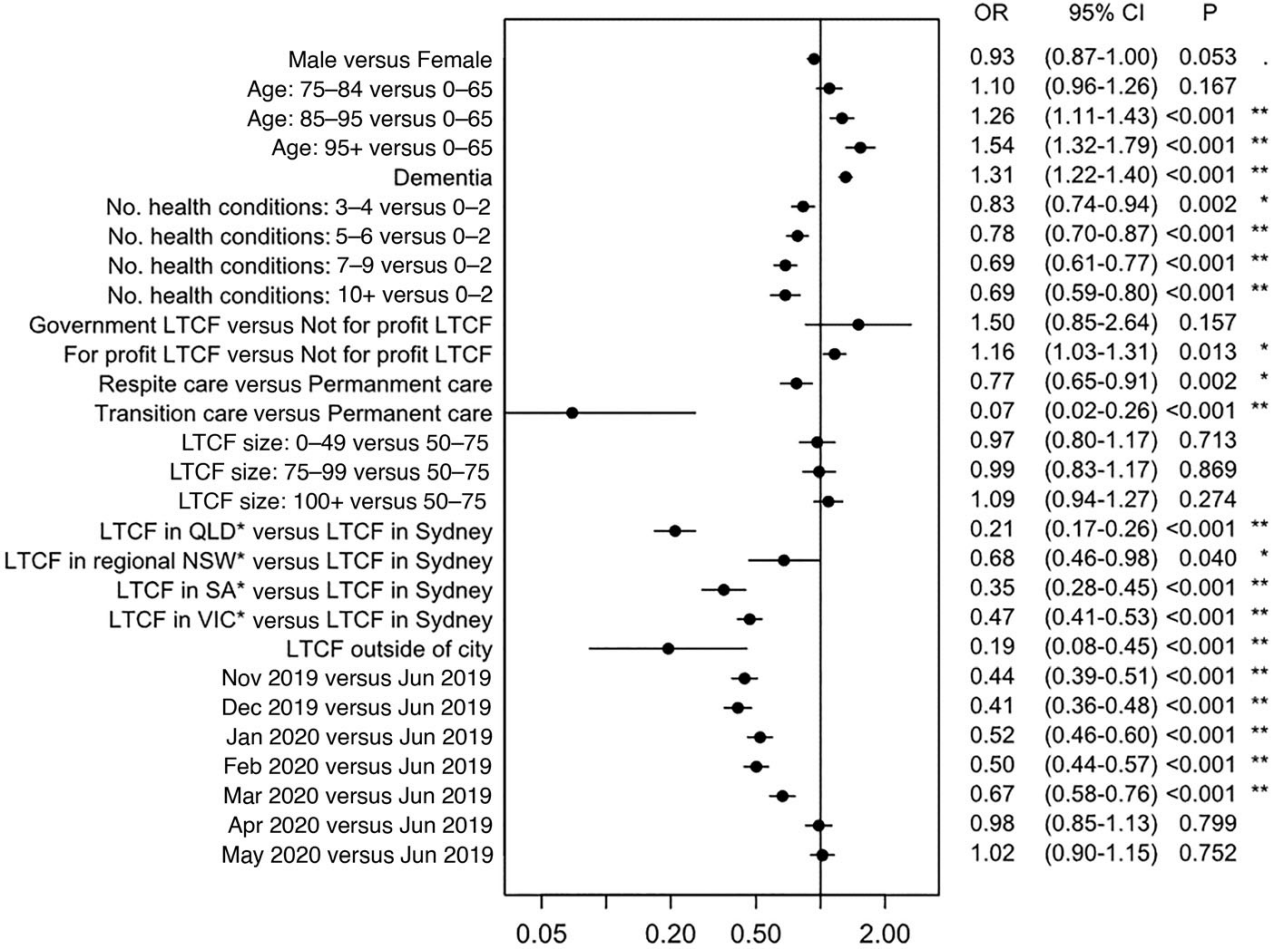
Associations of individual and long‐term care facility characteristics with mobile X‐ray services utilisation compared to community‐based X‐rays. *n* = 28 861 X‐ray events, of which *n* = 1760 (6.1%) were excluded due to missing data. LTCF, long‐term care facility; NSW, New South Wales; OR, odds ratio; QLD, Queensland; SA, South Australia; VIC, Victoria. *Only includes regions with access to mobile X‐ray services. Regional NSW = Illawarra and Shoalhaven region. VIC = greater Melbourne. QLD = greater Brisbane and Gold Coast; SA = greater Adelaide and Fleurieu–Kangaroo Island.

Compared to community‐based X‐ray recipients, residents accessing mobile X‐rays were older (OR = 1.10, 95% CI 0.96–1.26 for age 75–84 vs <65; OR = 1.26, 95% CI 1.11–1.43 for age 85–95 vs <65; OR = 1.54, 95% CI 1.32–1.79 for age 95+ vs <65), more likely to have a diagnosis of dementia (OR = 1.31, 95% CI 1.22–1.40) or be in a for‐profit LTCF (OR = 1.16, 95% CI 1.03–1.31), and residents were less likely to have a higher number of health conditions (OR = 0.83, 95% CI 0.74–0.94 for 3–4 vs ≤2 health conditions; OR = 0.78, 95% CI 0.70–0.87 for 5–6 vs ≤2 health conditions; OR = 0.69, 95% CI 0.61–0.77 for 7–9 vs ≤2 health conditions; OR = 0.69, 95% CI 0.59–0.80 for 10+ vs ≤2 health conditions) or be in transition care (OR = 0.07, 95% CI 0.02–0.26) or respite care (OR = 0.77, 95% CI 0.65–0.91). Residents of LTCF outside a major city (OR = 0.19, 95% CI 0.08–0.45), and all regions outside of Greater Sydney (see Fig. 2 for all estimates) were less likely to access mobile X‐rays. The sensitivity analysis excluding the dementia status variable did not change the parameter estimates presented (data not shown).

## Discussion

In this work, the resident and facility characteristics associated with the uptake of mobile X‐ray services in LTCFs in Australia were examined following the introduction of a federal subsidy to encourage the expanded use of these services. In the five geographical regions where the service was used, 3.7% of LTCF episodes accessed at least one X‐ray from a mobile service during this initial implementation of the subsidy compared with 9.6% accessing only community‐based X‐rays.

Within the five major geographical regions identified to have access to mobile X‐ray services, the utilisation of services increased by 75% between November 2019 and June 2020 but remained low (21.5% of eligible provisions of X‐ray services). This confirms our previous evaluation of the uptake of these services nationally using the publicly available MBS records.^16^ Additionally, this modest service uptake occurred despite the start of the COVID‐19 pandemic, which dramatically reduced the use of imaging services by LTCF residents and individuals in other settings (e.g. hospitals).^16^, ^17^ While mobile X‐ray service usage increased in May 2020, the percentage of imaging services that were mobile began to increase in March; the initial nationwide COVID‐19 lockdown commenced in March 2020.^18^ In prior qualitative studies, we examined the introduction of this mobile X‐ray subsidy nationally and have reported that generally residents, carers and healthcare and aged care providers are positive towards mobile X‐rays service utilisation.^19^, ^20^, ^21^ However, these studies identified several challenges preventing mobile radiology services from being the most beneficial to the resident population. For example, mobile radiology service providers reported encountering low demand, high business costs and workforce and geographical restrictions challenges.^20^ Residents, while recognising the potential cost–benefit of mobile radiology, highlighted issues of costs and payment processes leading to accessibility challenges.^21^ Informal carers (i.e. family members, next of kin) identified cost and a lack of availability awareness as the major factors hindering service uptake.^19^, ^20^, ^21^ Additionally, staff shortages and turnover are constant elements considered to contribute to service inaccessibility.^21^ While some of the challenges are likely surmountable, including changes in residents' expectations and lack of awareness, it is possible that some, including providers' cost considerations, workforce challenges and availability, which also affect many other areas in LTCFs, will require significantly higher investment.

Individuals in LTCFs have increasingly complex health and care needs^4^ and avoiding hospitalisations of these individuals is a clear challenge for health and aged care providers caring for these individuals. While few individuals accessed mobile X‐rays, those who did were more likely to be people at higher risk of adverse events associated with hospitalisations, including those with dementia and/or from older age groups.^10^ Additionally, those who had few health conditions were more likely to receive mobile X‐rays. These findings suggest that individuals for whom these services were generally intended (i.e. those who can wait for imaging services in the LTCFs) benefited from access. For‐profit LTCFs were more likely to enable service access than not‐for‐profit LTCFs. Residents of not‐for‐profit LTCFs are also less likely to be partially or fully financially supported by the Australian Government (38% of for‐profit vs 47% in not‐for‐profit vs 57% in government LTCFs)^5^ and may have greater ability to personally cover additional costs, such as mobile radiology services, consistent with the difference in uptake between the types of LTCFs. People in permanent care were more likely to access services compared to shorter‐term stays (respite or transition care). Residents in respite or transition care are generally more likely to have been medically assessed and have had diagnostic imaging examinations prior to entering the LTCF, which may partially explain this trend. Both factors are likely the result of models of care in place for these LTCFs and types of residents. Finally, in Sydney, NSW, where access to mobile X‐ray providers has been long established (i.e. one provider for >25 years), residents more frequently access these services, possibly due to greater service availability and awareness, demonstrating that other regions could improve their service access and processes.

Our study has several limitations, including the evaluation of only federally subsidised X‐ray use (and not wider availability) in LTCFs or the community. Our study is restricted to the geographical areas where individuals received mobile X‐ray services during the study period. Our study excluded data from Indigenous Australians who make up approximately 2% of the general residential care population, as we did not have the required Indigenous leadership, governance and ethics approval for this specific analysis. We have only examined variables in the ROSA to identify the factors associated with service provision. Other factors potentially associated with service utilisation, including service availability, care models and acceptability, were not examined. Finally, our study covered only the introductory months of these subsidies nationally, and it is possible that residents using them have changed.

Our study's strengths include the full coverage of LTCF residents in the regions included, which means these findings are generalisable to most LTCF residents of similar geographical areas. It also used an integrated data source that allows the evaluation of all government subsidised healthcare service utilisation by individuals in LTCFs, providing full capture of individuals in this setting and a low likelihood of loss to follow up and missing information. Finally, this study's variables have high internal validity, having been constructed using multiple data sources.

Our analysis of the five major geographical areas with 1449 LTCFs that have adopted the use of mobile X‐ray services has found a small increase in the use of the government subsidies over the first 8 months of the subsidy introduction. Given the significant increase in utilisation and growing investment in virtual care, geriatric outreach services and other care models that keep individuals out of hospital, mobile X‐rays are likely to contribute to the changing landscape of care for older people in LTCFs. Individuals accessing the services thus far are those that likely could benefit from them the most, which is encouraging, but their low use and LTCF differences in service utilisation indicate potential gaps in service access and use nationally.

## Supporting information


**Data S1.** Supporting Information.
