# Supplementary material for: Utilisation of mobile X‐ray services by residents of long‐term care facilities
Source: Intern Med J. 2025 Mar 24;55(6):951–8. doi: 10.1111/imj.70034 (PMC12155079; doi:10.1111/imj.70034)
Supplement: Supplementary file 1 — Data S1. Supporting Information. [file IMJ-55-951-s001.docx]

**Supplementary Files**

**Supplementary Table 1.** Medicare Benefits Schedule Items for X-ray Services^1^

| Mobile X-ray Item (57541) Eligible X-ray Classes | Medicare Benefits Schedule Items |
| --- | --- |
| Fall | 57509, 57515, 57521, 57527, 57530*, 57533*, 57539*, 57703, 57705*, 57709, 57711*, 57712, 57714*, 57715, 57717*, 58521, 58523*, 58524, 58526*, 58527, 58529*, 57536* |
| Pneumonia/Heart Failure | 58503, 58505* |
| Acute abdomen/bowel obstruction | 58903, 58905* |
|  |  |
| Body parts | Medicare Benefits Schedule Items |
| Chest | 58500, 58502, 58503^#^†, 58505^#^†, 58509, 58511, 58521^#^, 58523^#^, 58524^#^, 58526^#^, 58527^#^, 58529^#^ |
| GI tract plain abdominal | 58900, 58902, 58903^#^‡, 58905^#^‡ |
| Extremities / upper body including arms | 57506, 57509^#^, 57512, 57515^#^, 57529, 57530^#^, 57532, 57533^#^, 57000, 57702, 57703^#^, 57705^#^, 57706, 57708, 57709, 57711 |
| Extremities / legs | 57518, 57521^#^, 57535, 57536^#^, 57524, 57527^#^, 57538, 57539^#^, 57522, 57523, 57537, 57540 |
| Extremities / hip join and pelvis | 57712^#^, 57714^#^, 57715, 57717^#^ |
| Spine | 58100, 58103, 58106, 58109 |
| Head | 57945, 57956, 57901, 57907, 57915 |

* Eligibility for item ceased 30/4/2020.

^#^ Most relevant to falls.

† Code for pneumonia/heart failure.

‡ Codes for acute abdomen/bowel obstruction.

1. Notes: Table shows Medicare Benefits Schedule items that are eligible to be claimed with the mobile X-ray item and items for X-rays considered in our study. These other X-rays were provided concurrently with eligible mobile X-ray services.

**Supplementary Table 2**. Long-term care facility characteristics according to mobile X-ray service callouts (57541 item), Nov 2019 – Jun 2020.

|  |  |  | No | 57541 | Any | 57541 |
| --- | --- | --- | --- | --- | --- | --- |
|  |  |  | N | % | N | % |
| Cohort | 1449 | 100.0 | 492 | 34.0 | 957 | 66.0 |
| LTCF funding |  |  |  |  |  |  |
| Not-for-profit | 65 | 4.5 | 47 | 9.6 | 18 | 1.9 |
| Private | 731 | 50.4 | 262 | 53.3 | 469 | 49.0 |
| Government | 653 | 45.1 | 183 | 37.2 | 470 | 49.1 |
| LTCF places* |  |  |  |  |  |  |
| 0-24 | 36 | 2.5 | 24 | 4.9 | 12 | 1.3 |
| 25-49 | 299 | 20.6 | 140 | 28.5 | 159 | 16.6 |
| 50-74 | 391 | 27.0 | 140 | 28.5 | 251 | 26.2 |
| 75-99 | 298 | 20.6 | 95 | 19.3 | 203 | 21.2 |
| 100+ | 425 | 29.3 | 93 | 18.9 | 332 | 34.7 |
| State* |  |  |  |  |  |  |
| New South Wales – Greater Sydney | 477 | 32.9 | 88 | 17.9 | 389 | 40.6 |
| New South Wales – other | 52 | 3.6 | 28 | 5.7 | 24 | 2.5 |
| Victoria | 480 | 33.1 | 148 | 30.1 | 332 | 34.7 |
| Queensland | 257 | 17.7 | 139 | 28.3 | 118 | 12.3 |
| South Australia | 183 | 12.6 | 89 | 18.1 | 94 | 9.8 |
| LTCF remoteness |  |  |  |  |  |  |
| Major City | 1375 | 94.9 | 427 | 86.8 | 948 | 99.1 |
| Not Major City | 74 | 5.1 | 65 | 13.2 | 9 | 0.9 |

LTCF= long-term care facility.

* Only includes regions with access to mobile X-ray services.
